# Longitudinal Serology of SARS-CoV-2-Infected Individuals in India: A Prospective Cohort Study

**DOI:** 10.4269/ajtmh.21-0164

**Published:** 2021-05-18

**Authors:** Ramachandran Thiruvengadam, Souvick Chattopadhyay, Farha Mehdi, Bapu Koundinya Desiraju, Susmita Chaudhuri, Savita Singh, Vandita Bhartia, Pallavi Kshetrapal, Uma Chandra Mouli Natchu, Nitya Wadhwa, Shailaja Sopory, Mudita Wahi, Anil K. Pandey, Juhi Taneja, Nidhi Anand, Nandini Sharma, Pragya Sharma, Sonal Saxena, Deepa Sindhu, Brahmdeep Sindhu, Dharmendra Sharma, Tripti Shrivastava, Arjun Dang, Gaurav Batra, Gagandeep Kang, Shinjini Bhatnagar, for the DBT India Consortium for COVID 19 Research

**Affiliations:** 1 Translational Health Science and Technology Institute, Faridabad, Haryana, India;; 2 St. John’s Medical College and St. John’s Research Institute, Bengaluru, India;; 3 ESIC Medical College and Hospital, Faridabad, Haryana, India;; 4 Maulana Azad Medical College and Lok Nayak Hospital, New Delhi, India;; 5 Civil Hospital, Gurugram, India;; 6 Civil Hospital, Palwal, India;; 7 Dr. Dang’s Laboratory, New Delhi, India;; 8 Christian Medical College, Vellore, India

## Abstract

Clinical and epidemiological characteristics of severe acute respiratory syndrome coronavirus 2 (SARS-CoV-2) are now widely available, but there are few data regarding longitudinal serology in large cohorts, particularly those from low-income and middle-income countries. We established an ongoing prospective cohort of 3,840 SARS-CoV-2-positive individuals according to RT-PCR in the Delhi-National Capital Region of India to document clinical and immunological characteristics during illness and convalescence. The immunoglobulin G (IgG) responses to the receptor binding domain (RBD) and nucleocapsid were assessed at 0 to 7 days, 10 to 28 days, and 6 to 10 weeks after infection. The clinical predictors of seroconversion were identified by multivariable regression analysis. The seroconversion rates during the postinfection windows of 0 to 7 days, 10 to 28 days, and 6 to 10 weeks were 46%, 84.7%, and 85.3%, respectively (*N* = 743). The proportion with a serological response increased with the severity of coronavirus disease 2019 (COVID-19). All participants with severe disease, 89.6% with mild to moderate infection, and 77.3% of asymptomatic participants had IgG antibodies to the RBD antigen. The threshold values for the nasopharyngeal viral RNA RT-PCR of a subset of asymptomatic and symptomatic seroconverters were comparable (*P* = 0.48) to those of nonseroconverters (*P* = 0.16) (*N* = 169). This is the first report of longitudinal humoral immune responses to SARS-CoV-2 over a period of 10 weeks in South Asia. The low seropositivity of asymptomatic participants and differences between assays highlight the importance of contextualizing the understanding of population serosurveys.

## INTRODUCTION

Coronavirus disease 2019 (COVID-19), which is caused by severe acute respiratory syndrome coronavirus 2 (SARS-CoV-2), has emerged as one of the most significant global public health challenges of the 21st century. The clinical phenotype of SARS-CoV-2 infection varies across a spectrum ranging from asymptomatic to mild (moderate) illness to severe COVID-19. The proportions of the specific phenotypes differ with geographical location.^1^ Prospectively followed cohorts of SARS-CoV-2-infected individuals have provided the opportunity to accurately describe the clinical characteristics of the disease in diverse geographical settings during different time periods of evolution of the pandemic. When combined with a systematic collection of biospecimens, such cohorts can serve as useful platforms to answer the emerging questions of importance to public health.

The characterization of immunological responses to SARS-CoV-2 infection and its association with the clinical spectrum of disease is still evolving. Detection of anti-SARS-CoV-2 antibodies targeting nucleocapsid (NC) and spike protein, particularly the receptor binding domain (RBD) of the S protein, is being used to evaluate serological humoral responses according to age and clinical phenotypes. Data regarding the persistence of antibodies against these proteins are emerging, but few studies have been performed in low-income and middle-income countries.

We measured immunoglobulin G (IgG) antibodies against SARS-CoV-2 RBD and NC protein in participants enrolled in a multihospital-based prospective cohort from northern India for up to at least 6 weeks postinfection and correlated the clinical and demographic differences between seroconverters and nonseroconverters.

## METHODS

### Study design and participants.

This study was developed by interdisciplinary research institutes and hospitals in the National Capital Region of India. It was coordinated by the Translational Health Science and Technology Institute. The main clinical sites were ESIC Medical College Hospital, Faridabad, and Loknayak Hospital, New Delhi. The study protocol was approved by the Institute Ethics Committees of all participating institutions.

### Cohort enrollment.

During the ongoing cohort study, we enrolled COVID-19-positive patients across all ages tested at or admitted to designated COVID-19 testing centers or hospitals within 5 days of their positive RT-PCR test results. The eligibility criteria were positive RT-PCR results according to the National Testing Strategy of India and written informed consent.^2^ The strategy for including participants from testing centers involved the enrollment of outpatients; however, in-patient recruitment was performed in hospital wards was to enrich the cohort with patients with COVID-19.

### Follow-up.

The follow-up visits were designed to capture the clinical outcomes of illness (10–28 days after being diagnosed with SARS-CoV-2) and the early (6–10 weeks) and late (6 and 12 months) convalescent periods (Supplemental Figure 1). The duration of illness was defined as the date of onset of symptoms for symptomatic participants; it was defined as the date of positive tests results indicating SARS-CoV-2 infection among those who were asymptomatic.^3^ This report presents only data until early convalescence; later follow-up is still ongoing.

### Clinical data and biospecimen collection.

A trained research team collected clinical data and biospecimens at enrollment and follow-up. The demography and clinical characteristics focused on symptoms, comorbidities, drug history, and treatment details were collected by electronic data capture based on the standard operating protocol developed *a priori*. Venous blood samples were collected and transported according to biosafety protocols recommended by the Government of India and the World Health Organization.^4^ Serum was separated and stored in the biorepository at Translational Health Science and Technology Institute for subsequent analyses.

### Serological assays.

Anti-SARS-CoV-2 RBD IgG antibody was detected using an ELISA as described previously.^5^ Briefly, samples and controls were diluted to 1:50 in the sample diluent, and 100 μL was added per well of the stabilized RBD-coated plate. After 30 minutes of incubation at room temperature (23 ± 2°C), the plates were washed six times with wash buffer. Then, 50 μL of ready-to-use conjugate (horseradish peroxidase-labeled goat anti-human IgG Fcγ-specific antibody) was added to each well and incubated for 30 minutes at room temperature, followed by six washes. Then, 100 μL of substrate was added to each well and incubated at room temperature for 10 minutes; the reaction was stopped with 100 μL of stop solution in each well. The absorbance was measured on a microplate reader at 450 nm with 650 nm as a reference wavelength. The pooled negative control and pooled positive control were performed on each plate. The cutoff for each plate was calculated by taking the average optical density of the triplicate of the negative control and adding 0.2 to this value. The signal-to-cutoff ratio (S/Co) was calculated as the ratio of the optical density value from the test sample to the cutoff value. An S/Co ratio ≥ 1 was considered positive.

The anti-NC IgG chemiluminescence assay (SARS-CoV-2 IgG; Abbott Diagnostics Division, Sligo, Ireland) was performed according to the manufacturer’s instructions; a calibrator was used and positive and negative controls were performed before each batch of antibody testing according to the manufacturer’s protocol. The results were obtained by dividing the chemiluminescent signal from the sample by the mean of chemiluminescent signals from three calibrator replicates. The default result unit is index (S/Co); results higher than a cut-off of 1.4 were considered positive.

### Statistical analysis.

Seroconverters were defined as those whose test results were seropositive according to either assay at least once any time during the study period comprising 10 weeks of follow-up. The clinical phenotypes of COVID-19 were stratified as severe, mild to moderate, and asymptomatic based on their most severe symptoms and/or the need for treatment at enrollment or at any time during the follow-up period. Participants were considered to have severe COVID-19 if they died or required oxygen supplementation or ventilatory support, or if they required treatment in the intensive care unit for cardiopulmonary or multiorgan dysfunction. Mild to moderate disease was defined by the presence of symptoms of COVID-19 that did not fulfill the criteria for severe disease. Those who did not report any symptoms at enrollment or throughout the follow-up period were classified as having asymptomatic COVID-19. The clinical and epidemiological characteristics of the participants are described as median and interquartile range (IQR) for continuous variables, and as percentages for categorical variables. The univariate analyses were performed using the χ^2^ test for comparison of proportions. The Mann Whitney *U* test was performed to determine the differences in distributions between among clinical groups (seroconverters and nonseroconverters). A multivariable regression analysis was performed to identify independent predictors of seroconversion. We included all clinical variables collected during the study as independent variables in the multivariable regression analysis. The differences in cycle threshold values between asymptomatic and symptomatic individuals in both seroconversion and nonseroconversion categories were tested for significance using the Mann Whitney *U* test. All statistical analyses were performed using R.

## RESULTS

### Baseline and clinical characteristics of the participants.

The cohort had 3,790 COVID-19 patients on December 18, 2020; the majority of those enrolled were from Lok Nayak Hospital, New Delhi (45%) and ESIC Medical College Hospital, Faridabad (44.6%). We present the clinical results of the first 2,504 (2139 from the hospital and 365 from testing centers) participants enrolled between April 2020 and October 2020, that were obtained 6 to 10 weeks postdiagnosis. The median age was 44 years (IQR, 30–57), and more than two-thirds of the patients were male. Nearly two-thirds of the patients presented with symptoms; the most common symptoms were fever (61%), cough (48%), breathlessness (35%), sore throat (27%), and body ache (22%). Only one out of five individuals had a history of primary contact defined as direct contact for 15 minutes without a mask with someone who had positive test results for COVID-19. A history of secondary contact, defined as direct contact for 15 minutes without a mask with someone who had a history of primary contact was noted for 2%. Among those with a history of contact, the median duration since contact was 4 days (IQR, 2–7 days). The baseline characteristics of the participants who underwent serological analysis (*N* = 743) are provided in Table 1.

**Table 1:** Demographic and clinical characteristics of participants

	Full cohort (*N* = 2,504)		Participants included for serological evaluation (*N* = 743)
Baseline characteristics	N	Median (IQR or IQR; 95% CI) or n (% or %; 95% CI)	Median (IQR or IQR; 95% CI) or n (% or %; 95% CI)
Age, years	2,452	42 (29–55)	40 (29–53)
Male	2,504	1,703 (68%)	519 (70%)
Symptomatic	2,504	2,078 (83%)	626 (84%)
Healthcare workers	2,504	257 (10.3%)	57 (7.3%)
History of primary contact	2,504		
No		1,510 (60%)	441 (59%)
Not known		483 (19%)	139 (19%)
Yes		511 (20%)	163 (22%)
History of secondary contact	2,504		
No		1,551 (62%)	447 (60%)
Not known		889 (35%)	275 (37%)
Yes		64 (2.6%)	21 (2.8%)
Duration of contact, days	386	4.0 (2.0–7.8)	4.0 (2.0–8.0)
Duration of contact, hours per day	309	12 (6–24)	12 (6–24)
Outcome characteristics			
Disease severity	2,504		
Asymptomatic		411 (16%; 14.98–17.92)	199 (25.5%; 22.42–28.65)
Mild to moderate		1,638 (65%; 63.51–67.27)	513 (65.6%; 62.15–68.93)
Severe		455 (18%; 16.67–19.73)	70 (8.95%; 7.0–11.17)
Intensive care	2,278	75 (3.3%; 2.59–4.10)	24 (3.1%; 1.98–4.53)
Oxygen therapy	2,313	432 (19%; 17.10–20.32)	134 (17%; 14.55–19.96)
Mechanical ventilation	2,275	10 (0.4%; 0.21–0.80)	2 (0.3%; 0.03–0.92)
Death	2,504	23 (0.9%; 0.58–1.37)	0 (0%)
Time to death from date of diagnosis, days	23	25 (16–35)	NA
Time to death from date of onset of symptoms, days	20	34 (20–43)	NA

Participants were considered to have severe COVID-19 if they died or required oxygen supplementation or ventilatory support, or if they required treatment in the intensive care unit for cardiopulmonary or multiorgan dysfunction. Mild to moderate disease was defined by the presence of symptoms of COVID-19 that did not fulfill the criteria for severe disease. Those who did not report any symptoms at enrollment or throughout the follow-up period were classified as having asymptomatic COVID-19. CI = confidence interval; IQR = interquartile range.

### Serological characteristics.

**Figure 1.**
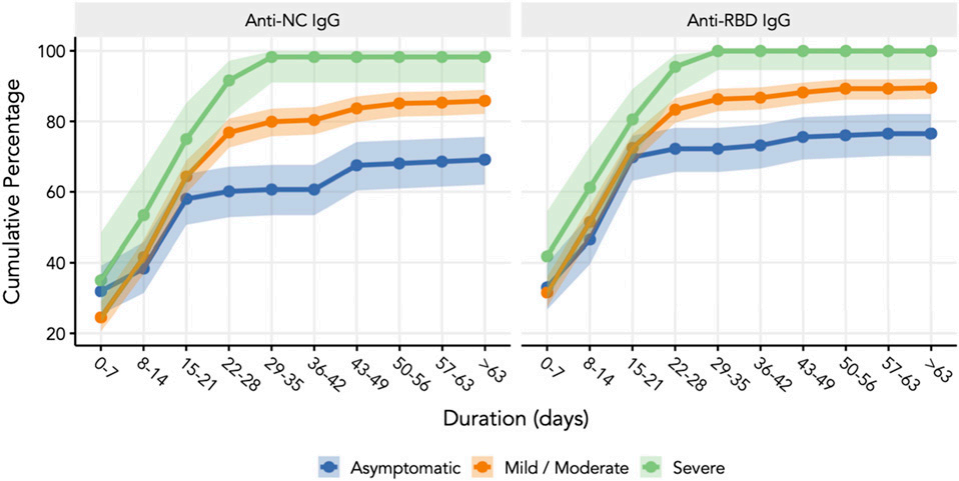
Cumulative seropositivity for anti-nucleocapsid (anti-NC) and anti-receptor binding domain (anti-RBD) immunoglobulin G (IgG) antibodies among different categories of disease severity. The error bars indicate the 95% confidence interval (CI) of the signal/cutoff ratio during that window of follow-up. This figure appears in color at www.ajtmh.org.

**Figure 2.**
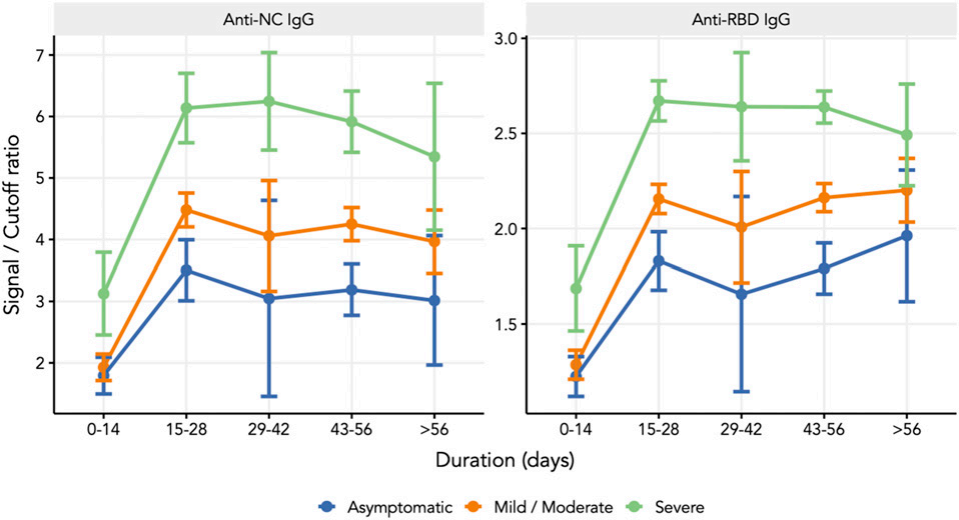
Longitudinal changes in the signal to cut-off ratios among different categories of disease severity. Anti-NC = anti-nucleocapsid; anti-RBD = anti-receptor binding domain; IgG = immunoglobulin G. This figure appears in color at www.ajtmh.org.

The longitudinal serological evaluation included participants who contributed all three samples (*N* = 743). The seroconversion rates during the windows of 0 to 7 days, 10 to 28 days, and 6 to 10 weeks were 46%, 84.7%, and 85.3%, respectively (Table 2) and Supplemental Table 1. The positive proportion increased with the severity of COVID-19; 100% with severe disease, 89.6% with mild to moderate disease, and 77.3% who were asymptomatic had IgG antibodies to the RBD antigen. As shown in Figure 1 and Table 3, the response to the NC antigen by the anti-NC chemiluminescence assay was lower (severe, 98.3%; mild–moderate, 85.9%; and asymptomatic, 69.9%). Higher seroconversion with disease severity was consistent when we evaluated the S/Co ratio of the anti-RBD antibody and the anti-NC IgG antibody (Figure 2). Notably, those who were seropositive for anti-RBD IgG at enrollment had an almost 60% higher risk of severity (38/242 versus 29/294; relative risk [RR], 1.59; 95% confidence interval [CI], 1.01–2.50) than those who remained seronegative or became seropositive at a later time point. The threshold values of the nasopharyngeal viral RNA RT-PCR of asymptomatic and symptomatic seroconverters were comparable (*P* = 0.48), as were those of asymptomatic and symptomatic nonseroconverters (*P* = 0.16) (*N* = 169) (Supplemental Figure 2). For all those who had an IgG response, there was no demonstrable decline, as seen by the S/Co ratio during up to 10 weeks of follow-up.

**Table 2:** Seropositivity for antibodies against different antigens (*N* = 672)

	Positive only for anti-RBD IgG, n (%)	Positive only for anti-NC IgG, n (%)	Positive for both antibodies, n (%)	Positive for either of the antibodies, n (%)
Within 7 days of illness	76 (11.3%) (95% CI, 9.01–13.95)	31 (4.6%) (95% CI, 3.15–6.48)	202 (30.1%) (95% CI, 26.61–33.68)	309 (46.0%) (95% CI, 42.16–49.83)
By 10–28 days of illness	56 (8.3%) (95% CI, 6.35–10.68)	10 (1.5%) (95% CI, 0.71–2.72)	503 (74.9%) (95% CI, 71.39–78.09)	569 (84.7%) (95% CI, 81.72–87.31)
By 6–10 weeks of illness	45 (6.7%) (95% CI, 4.92–8.85)	4 (0.6%) (95% CI: 0.16–1.52)	523 (77.8%) (95% CI, 74.49–80.91)	573 (85.3%) (95% CI, 82.36–87.86)

Seropositivity is presented as absolute numbers and percentages. This analysis included 672 participants who had results of both anti-RBD and anti-NC assays across three time-points. anti-NC = anti-nucleocapsid; anti-RBD = anti-receptor binding domain; CI = confidence interval; IgG = immunoglobulin G.

**Table 3:** Proportion of seroconverters among different categories of disease severity

Type of assay	Asymptomatic, n (%)	Mild to moderate, n (%)	Severe, n (%)	Proportion among all categories, n (%)
Anti-RBD IgG (*N* = 743)	160 (77.29%) (95% CI, 70.98–82.81)	420; 89.55% (95%CI: 86.42–92.17)	67 (100.00%) (95% CI, 94.64– 100.00)	647 (87%) (95% CI, 84.45–89.41)
Anti-NC IgG (*N* = 673)	130 (69.89%) (95% CI, 62.75–76.39)	364 (85.85%) (95% CI, 82.16–89.02	59 (98.33%) (95% CI, 91.06–99.96)	553 (82.16%) (95% CI, 79.06–84.99)

The last column indicates the overall seropositivity rates. Numbers in the parentheses denote the 95% confidence intervals of the percentage estimates of seroconverters. Participants had results for both anti-RBD and anti-NC assays across categories of disease.

### Predictors of seroconversion.

Demographic and clinical characteristics associated with seroconversion are described in Table 4

**Table 4:** Demographic and clinical characteristics associated with seroconversion (*N* = 743)

Characteristic	Total N of participants	Proportion of seroconverters, n (%)	Unadjusted odds ratio (95% CI)	*P* value
Sex				
Female	212	176 (83.02%)	0.75 (0.47–1.2)	0.24
Male	513	445 (86.74%)		
Symptom status at enrollment				
Asymptomatic	122	83 (68.03%)		< 0.001
Symptomatic	603	538 (89.22%)	3.88 (2.38–6.29)	
Age				
0–30 years	190	143 (75.26%)		< 0.001
30–60 years	410	358 (87.32%)	2.26 (1.42–3.59)	
> 60 years	109	105 (96.33%)	8.58 (3.00–33.81)	
Comorbidities				
Asthma				
No	708	609 (86.02%)		0.15
Yes	17	12 (70.59%)	0.39 (0.12–1.45)	
Autoimmune disorders				
No	721	618 (85.71%)		1.00
Yes	4	3 (75%)	0.50 (0.04–26.5)	
Cancer				
No	715	612 (85.59%)		
Yes	10	9 (90%)	1.51 (0.21 67.01)	
Diabetes				
No	597	500 (83.75%)		< 0.001
Yes	128	121 (94.53%)	3.35 (1.51–8.77)	
Heart disease				
No	696	594 (85.34%)		0.37
Yes	29	27 (93.1%)	2.32 (0.57–20.4)	
Hypertension				
No	605	505 (83.47%)		< 0.001
Yes	120	116 (96.67%)	5.73 (2.10–21.89)	
Kidney disease				
No	717	616 (85.91%)		0.17
Yes	8	5 (62.5%)	0.27 (0.05–1.79)	
Liver disease				
No	717	615 (85.77%)		0.72
Yes	8	6 (75%)	0.50 (0.09–5.11)	
More than one comorbidity				
No	624	530 (84.94%)		0.22
Yes	101	91 (90.1%)	1.61 (0.80–3.61)	
Smoking				
No	667	574 (86.06%)		0.39
Yes	58	47 (81.03%)	0.69 (0.34–1.54)	
Thyroid disorders				
No	697	597 (85.65%)		1.00
Yes	28	24 (85.71%)	1.01 (0.34–4.07)	

The multivariable model including all the predictors listed in this table showed that age (years) (adjusted odds ratio [aOR], 1.03; 95% confidence interval [CI], 1.02–1.05) and the presence of symptoms at presentation (aOR, 3.23; 95% CI, 2.01–5.17) were independent predictors of seroconversion.

. More than 95% (*N* = 105) of participants older than 60 years had positive IgG antibody results; this proportion decreased with younger age. Interestingly, participants who reported no primary contact or those who were uncertain about their contact history experienced seroconversion more often than those who had a history of exposure. Seroconverters reported a longer exposure duration per day than those who did not exhibit an IgG response (median exposure: 24 hours versus 8 hours) (Table 5

**Table 5:** Differences in the epidemiological characteristics of seroconverters and nonseroconverters

Baseline characteristics	History of contact, N	Nonseroconverters, median (IQR)	Seroconverters, median (IQR)	*P* value
Duration of contact, days	124	4.0 (1.0–8.5) (*N* = 31)	4.0 (2.0–7.0) (*N* = 93)	0.8
Duration of contact, hours per day	99	8 (2–24) (*N* = 27)	24 (7–24) (*N* = 72)	0.01
Duration between date of first contact and date of diagnosis, days	127	9 (5–11) (*N* = 33)	6 (3–10) (*N* = 94)	0.13
Duration between date of last contact and date of diagnosis, days	122	1.0 (−1.0 to 6.0) (*N* = 31)	1.0 (−1.0 to 4.0) (*N* = 91)	0.6

The history of contact was available for 185 of 743 participants. IQR = interquartile range.

). The presence of pre-existing comorbidities such as chronic hypertension (*P* < 0.001) and diabetes mellitus (*P* < 0.001) were associated with seroconversion (Table 4). The multivariable model after adjusting for confounders indicated that age (years) (adjusted odds ratio [aOR], 1.03; 95% CI, 1.02–1.05) and the presence of symptoms at presentation (aOR, 3.23; 95% CI, 2.01–5.17) were independent predictors of seroconversion.

## DISCUSSION

Age and symptomatic status were independent positive predictors of seroconversion for our Indian cohort. The higher antibody response with severe COVID infection was expected. A severe infection indicated either a higher infectious dose or the spread of viruses beyond the respiratory tract. In both situations, the probability of the host immune response increases.^6–9^ Furthermore, longer exposure of the host immune system caused by prolonged severe COVID-19 infection may lead to the generation of polyfunctional T cells, which elicit a cytokine storm and an evident humoral response.^10,11^ Our findings differ from those of studies performed in western countries that showed much higher seroconversion rates among asymptomatic individuals.^12–14^ However, a recent study performed in Bangladesh reported lower seroconversion rates (45%) among asymptomatic individuals.^15^ This raises the possibility that biological responses to SARS COV-2 may vary among different populations.

Age is a known strong predictor of severe COVID-19, and it is possible that systemic spread of infection, possibly because of impaired innate immune protection, occurs in elderly individuals. The presence of comorbidities, such as chronic hypertension and diabetes mellitus, was found to be associated with seroconversion by the unadjusted analysis; this relationship was probably confounded by the ages of the participants.

Nearly 15% of participants did not have IgG antibodies against both RBD and NC antigens. The trends of seropositivity were largely similar; anti-RBD IgG were detectable marginally earlier during the course of illness and in a larger proportion of individuals than anti-NC IgG. This was particularly noticeable among asymptomatic individuals. There could be many reasons for the poorer antibody response among asymptomatic individuals in our cohort. Unlike with severe infection, a possible lack of systemic spread of the virus in asymptomatic individuals would result in less exposure of the immune system. A large proportion of these individuals were identified by active contact tracing, and no or low seropositivity could reflect less exposure to a SARS-CoV-2-positive contact.^16^ It has been shown that the nasopharyngeal viral load is significantly higher among individuals with severe COVID-19 compared with that of individuals with mild illness.^17^ A small subset of individuals were seronegative until day 21 of their illness; however, they were seropositive at 6 to 10 weeks postinfection. This “negative-negative-positive” group of participants did not report any symptoms suggestive of SARS-CoV-2 infection; however, the possibility of having acquired a second asymptomatic infection during the follow-up period cannot be ruled out.

The lower seroconversion rates among asymptomatic RT-PCR-positive participants are of critical significance to public health, particularly for the interpretation of community seroprevalence in highly affected geographical areas. Results of seroprevalence studies performed in India and other South Asian countries may need to be adjusted after considering our findings.^18,19^

The S/Co ratio for both assays reached the maxima between 15 and 28 days and plateaued, suggesting a stable IgG response during up to 10 weeks of illness. The longevity of the IgG response has been reported to vary between 5 weeks to 4 months.^13,20–24^ As shown by another global study, asymptomatic participants in our study did not show any decline in the IgG response to either antigen during a period of 10 weeks.^25^ This is in contrast to the initial evidence that showed early decline in the IgG response in asymptomatic infected individuals.^3,13^ We will follow-up the participants in this cohort for at least 1 year to evaluate the longevity of the IgG response.

The major strengths of the study are the prospective data collection, high follow-up rates, and the use of two validated antibody assays. However, there are some limitations. Because of the lack of robust IgM and IgA assays, we were unable to evaluate the immune response to these isotypes. Furthermore, because of the heterogeneity of the RT-PCR assays used by the participating hospitals for molecular diagnosis, we do not have comparable viral load data for all participants in our study. However, an analysis of a subset of the individuals who had similar RT-PCR assay results showed no differences in nasopharyngeal viral loads between seroconverters and nonseroconverters. The 95% CI for the association between the presence of anti-RBD IgG antibodies at enrollment and the severity of COVID-19 is wide, with the lower bound of the interval close to 1, suggesting the need for a larger sample size to confirm the association.

We systematically reported clinical and epidemiological characteristics of and longitudinal humoral immune responses to SARS-CoV-2 infection by a large Indian cohort. We will continue to study the kinetics of the immune response in a unique platform to evaluate the more complex and emerging questions regarding cellular immunity, reinfections, long COVID syndrome, and population-level postimmunization surveillance when the cohort participants are immunized.

## Supplemental information, tables, and figure

Supplemental materials
