# Supplementary material for: Longitudinal Serology of SARS-CoV-2-Infected Individuals in India: A Prospective Cohort Study
Source: Am J Trop Med Hyg. 2021 May 18;105(1):66–72. doi: 10.4269/ajtmh.21-0164 (PMC8274753; doi:10.4269/ajtmh.21-0164)
Supplement: Supplementary file 1 [file tpmd210164.SD1.pdf]

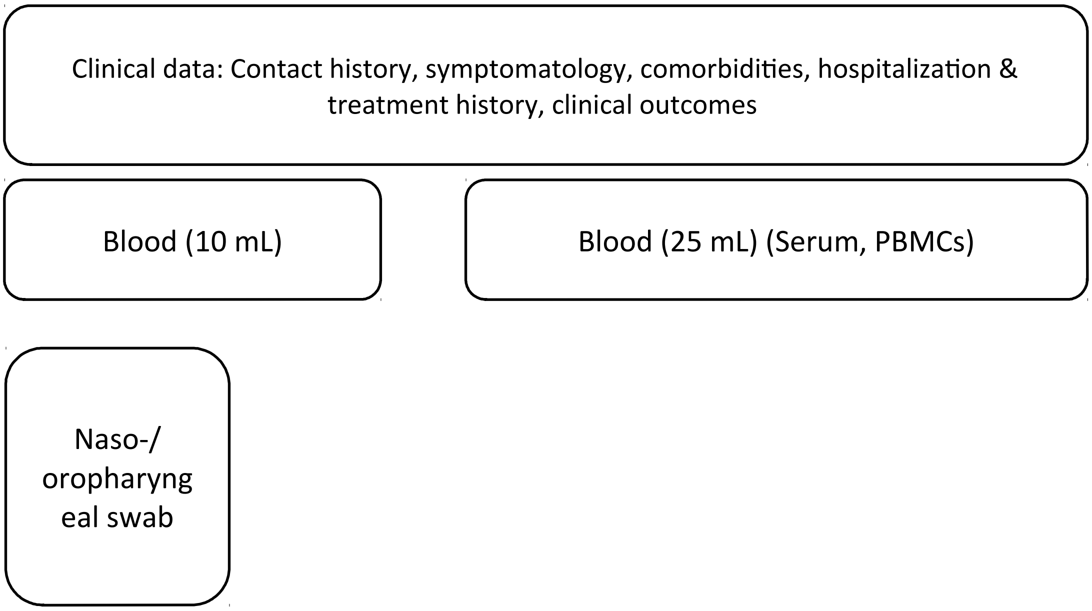

Supplemental Figure 1. Study flow of the cohort. The follow up presented here is up to 6-10 weeks.

Supplemental Figure 2. Comparison of RT-PCR threshold values (CT) between asymptomatic and symptomatic participants among seroconverters and non-seroconverters (N=169).

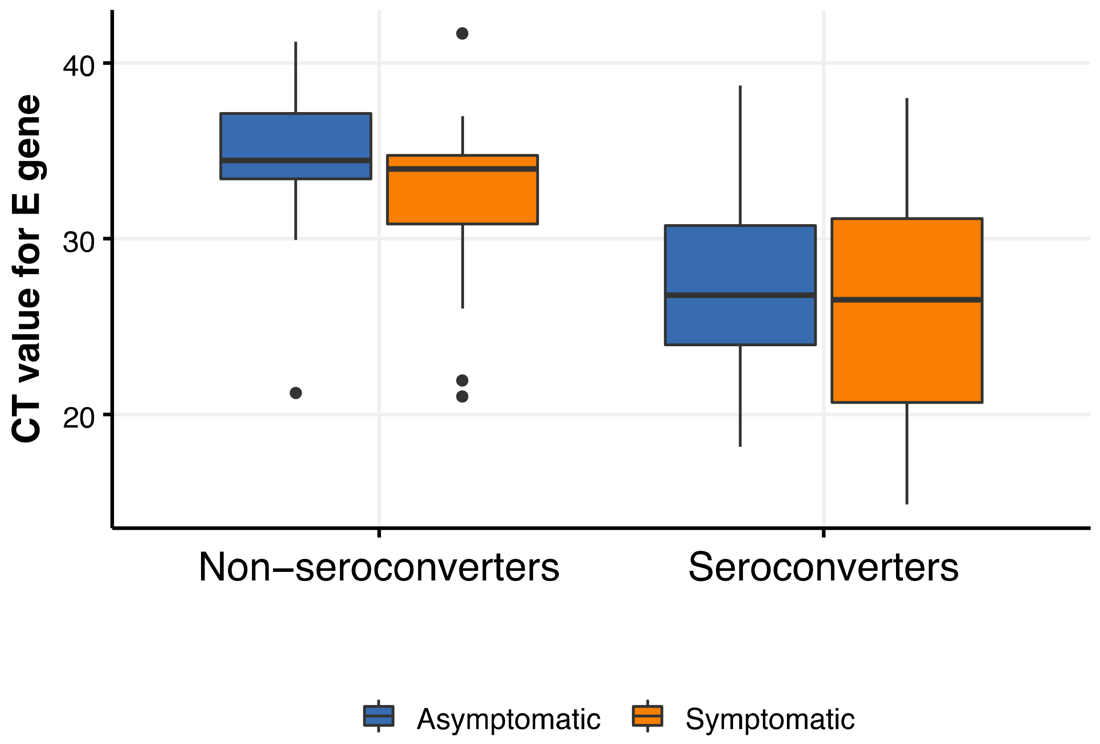

False: Non seroconverters; True: Seroconverters. Statistical test applied between asymptomatic and symptomatic groups: Mann Whitney test; among seroconverters: p-value 0.48 and among non seroconverters: *P*-value 0.16.

Supplemental Table S1. Patterns of seropositivity across the three time-windows of illness

Within 7 days of onset of illness*	10-28 days	6-10 weeks	Anti-RBD assay [n (%)] N=743	Anti-NC assay [n (%)] N=673
Negative	Negative	Negative	99 (13.3)	120 (17.8%)
Negative	Negative	Positive	21 (2.8)	39 (5.8%)
Negative	Positive	Negative	7 (1.0)	12 (1.8%)
Negative	Positive	Positive	305 (40.9)	269 (40.0%)
Positive	Negative	Negative	2 (0.3)	1
Positive	Negative	Positive	1 (0.1)	0
Positive	Positive	Negative	2 (0.3)	12 (1.9%)
Positive	Positive	Positive	309 (41.4)	220 (33.7%)

* From the onset of symptoms in case of symptomatic or date of testing for asymptomatic participants.

Anti-NC and anti-RBD IgG denote anti-nucleocapsid and anti-RBD Immunoglobulin-G respectively.
